# The Influence of Winning and Losing Gambling Experience on Mood State and Alcohol Cravings

**DOI:** 10.1007/s10899-024-10367-7

**Published:** 2024-12-14

**Authors:** Damon Lipinski, James P. Whelan, Blaine E. Stiglets, Matthew D. Andersland, Meredith K. Ginley, Rory A. Pfund

**Affiliations:** 1 Department of Pediatrics, University of Arkansas, Fayetteville, AR USA; 2 Department of Psychology, The University of Memphis, Memphis, TN USA; 3 The Institute for Gambling Education & Research, Tennessee, Memphis and Johnson City, TN USA; 4 Department of Psychology, East Tennessee State University, Johnson City, TN USA

**Keywords:** Gambling, Alcohol, Craving, Mood

## Abstract

Previous work has explored the bi-directional relation between alcohol consumption and gambling behavior within gambling contexts, highlighting the role of salient factors such as mood. The present experiment sought to further explore how mood state and the urge to consume alcohol vary as a function of the experience of winning or losing while gambling. In this experiment, 76 individuals who reported past year gambling and past month alcohol use were randomly assigned to one of four conditions – neutral gambling, winning gambling, losing gambling, or movie watching. Results indicated that mood state did not significantly differ by experimental condition. However, the urge to consume alcohol significantly differed by experimental condition when accounting for weekly alcohol consumption. Individuals who underwent a losing gambling experience reported significantly greater alcohol-related urges than those who watched a movie or had a neutral gambling experience, but not compared to those who had a winning gambling experience. Exploratory analyses showed that those exposed to a losing gambling experience wanted a significantly greater number of drinks than those who had a neutral gambling experience or watched a movie. These results suggest that experiencing losing may contribute to a greater desire to consume alcohol, and that this relation may occur independent of mood states associated with gambling outcomes. Future research should seek to investigate the relations between gambling, alcohol use, and mood in naturalistic gambling settings beyond the laboratory.

## Introduction

Alcohol consumption and gambling commonly co-occur (Barnes et al., 2015), and comorbid presentation of alcohol use disorder and gambling disorder is well documented (Armoon et al., 2023; Dowling et al., 2015; Lorains et al., 2011). Untangling the reciprocal relation between these behaviors has progressed, though many questions remain untested. Specifically, laboratory research has revealed how alcohol consumption appears to impact gambling behavior (Corbin & Cronce, 2017; Horn et al., 2022; Lipinski et al., 2023a). However, less is understood about how gambling influences the consumption of alcohol (Lipinski et al., 2023b; Tobias-Webb et al., 2019) and what contextual variables influence the relation between these two behaviors. The present study sought to explore how the outcome of a gambling experience impacts mood state and the urge to consume alcohol.

A meta-analysis of experimental studies investigating the effect of alcohol administration on gambling and risk-taking behaviors, found no effect of alcohol on gambling and risk taking (Horn et al., 2022). However, subgroup analyses revealed significantly larger effect sizes among studies using non-alcholoic beverage control conditions compared to placebo-alcohol controls. This finding highlights the role of psychological factors such as alcohol expectancies, beliefs regarding the expected effects of alcohol consumption on behavior, which are hypothesized to contribute to alcohol use behavior (see Jones et al., 2001 for a review). Experimental findings have supported the role of expectations in alcohol use within gambling contexts (Lipinski et al., 2023a) and cross-sectional methods have observed relations between alcohol expectancies and gambling and alcohol-related harm (Horn et al., 2023). Alcohol expectations initially develop through socio-environmental experiences (Smit et al., 2018) and continue to evolve following the initiation of alcohol use and interaction with contexts in which alcohol consumption occurs (Jester et al., 2015; Treloar Padovano et al., 2020). This progression suggests the potential for stimulus functions acquired by contextual features associated with alcohol consumption (e.g., the smell or taste of alcohol), contacted within situations that include gambling-related stimuli, to evoke gambling behaviors like those evoked by the alcohol consumption – highlighting one avenue through which gambling may influence alcohol use behavior.

Though the influence of gambling on alcohol consumption has received relatively less empirical attention, extant research has observed increases in alcohol consumption following gambling (Lipinski et al., 2023b; Tobias-Webb et al., 2019). In a study evaluating the effect of slot machine play on alcohol consumption, participants assigned to 30-minutes of slot play ordered more drinks, drank a larger quantity of alcohol, consumed the alcohol faster, and reported a greater intention to drink than those who watched a television show (Tobias-Webb et al., 2019). Lipinski and colleagues (2023b) randomly assigned participants to gamble, watch an exciting sporting event, watch a non-exciting sporting event, or watch a movie. Participants who gambled or watched an exciting sports event reported significantly greater urges to drink and a more positive mood state than those assigned to the non-exicting sporting event or movie watching conditions. This finding, alongside the observed correlations between state anxiety and vigor, highlighted the potential interplay of both positive and negative mood in the relation between gambling and alcohol use (Lipinski et al., 2023b).

Mood has been theorized to contribute to engagement in gambling behaviors (e.g., Blaszczynski & Nower, 2002). Contexts that elicit negative mood states may further contribute to gambling behavior within a gambling event. Participants who underwent a negative self-reflection task engaged in faster play, placed larger bets, and continued to gamble longer than those assigned to a control condition (Rockloff et al., 2011). Participants randomly assigned to view a sad movie clip showed greater gambling persistence than those assigned to a control condition (Devos et al., 2018). Mood states of both arousal and sadness have been positively associated with the desire to gamble (Quilty et al., 2017), although relations between emotional experience and gambling are mixed (Cummins et al., 2009; Juergensen et al., 2018; Mishra et al., 2010). Nascent evidence supports the potential of gambling to elicit positive mood states (Kruger et al., 2020; Lipinski et al., 2023b; Mishra et al., 2010). Furthermore, the outcomes of a gambling experience (i.e., winning or losing) may contribute to alterations in mood (Gee et al., 2005; Hills et al., 2001; Mishra et al., 2010). As such, gambling outcomes may present one mechanism by which mood state, gambling, and alcohol consumption are associated.

Links between mood state and alcohol use have been observed among various domains. In a meta-analysis examining same-day relations between alcohol use and mood state, same-day positive mood was associated with an increased likelihood of any alcohol use and an increased likelihood of binge drinking, although same-day negative mood was not (Dora et al., 2023). Negative mood state has, however, been related to alcohol use and craving within experimental contexts. A meta-analysis of laboratory studies evaluating the effect of negative mood state manipulations on alcohol observed significant increases in both alcohol use and craving (Bresin et al., 2018).

Events that elicit both positive and negative mood states may be related to engagement in both gambling and alcohol use. The present study was designed to explore how the experience of gambling – specifically having a winning, losing, or neutral experience while gambling – may contribute to the urge to drink alcohol as well as influence mood state. Research has concluded that both winning and losing can influence mood while gambling (Gee et al., 2005; Hills et al., 2001; Mishra et al., 2010) and higher levels of positive or negative mood state are related to greater alcohol consumption (Bresin et al., 2018; Dora et al., 2023). Together, this implies that a person may be more likely to experience urges to drink based on a winning or losing experience.

To further explore the differential effects of winning and losing gambling experiences on mood and alcohol use, the present study sought to experimentally manipulate the outcomes of a gambling experience and assess subsequent mood state and alcohol craving. Participants were randomly assigned to a losing, neutral, or winning gambling experience or the control experience of watching a movie. Watching a movie was selected as the control condition as it is a common recreational activity providing a neutral, non-gambling related experience to participants, similar to controls employed in previous research in this area (e.g., Lipinski et al., 2023b; Tobias-Webb et al., 2019).

The first hypothesis was that subjective anxiety/arousal scores and total mood disturbance would be greater among those who gambled compared to the movie watching condition, and that participants in the gambling loss condition would self-report higher subjective anxiety/arousal and mood disturbance scores compared to the neutral gambling and movie watching condition. Participants in the gambling win condition were expected to self-report higher vigor compared to those in the neutral gambling and movie watching condition. The second hypothesis was that those who gambled would have higher alcohol cravings than those in the movie watching condition. Individuals in the win and loss gambling conditions were expected to have higher alcohol cravings than those in the neutral gambling condition.

## Methods

### Participants

Eighty participants over the age of 18 years were recruited from psychology classes and flyers posted across campus at a major urban university in Tennessee, USA. To qualify, participants had to report gambling at least once in the past year, consuming at least three alcoholic drinks in the past month, and pass a screener to ensure the absence of a gambling problem. Qualifying participants (*N* = 76) were 65.8% female and 34.2% male. The average age of participants was 21.4 years (*SD* = 5.04). The sample was 56.6% African American, 3.9% Asian American, 35.5% White, 1.3% Native American, and 2.6% other. Participants reported gambling 19.8 times (*SD* = 65.8) in the past year and drinking an average of 2.0 (*SD* = 2.6) standard alcoholic beverages while gambling. Participants reported consuming 8.6 (SD = 8.0) drinks in an average week in the past 30 days. Past month binge drinking was reported by 66.0% of women and 60.7% of men. See Table 1 for additional demographic and gambling history information. Participants received extra credit for their psychology class and raffle tickets for prizes. Data is available on reasonable request.

**Table 1:** Characteristics of study participants (*N* = 76)

Variable	M (SD) or %
Age	21.43 (5.04)
Gender Female Male	65.8% 34.2%
Years of College Education Less than 1 1 2 3 4 or more	39.5% 10.5% 22.4% 13.2% 14.5%
Monthly Income (dollars)	989 (1004.00)
Race African American White Other	56.6% 35.5% 7.8%
Marital Status Never Married/Single Separated Married Divorced	90.8% 1.3% 3.9% 3.9%
Past year gambling frequency (number of times gambled)	19.83 (65.78)
Largest past year single bet (dollars)	138 (451.00)
Average time spent per gambling episode in past year (hours)	2.91(4.58)
% reporting drinking alcohol while gambling	N/A
Average number of standard drinks while gambling ^a^	2.01 (2.59)
AUDIT score	7.91 (5.90)
Number of standard drinks per typical week in the past 30 days	8.56 (8.00)
Number of standard drinks per heaviest week in the past 30 days	13.16 (12.48)

AUDIT: Alcohol Use Disorders Identification Test; ^a^ Standard Drink = A standard US drink is the equivalent of one 12 oz. can of beer, one 5 oz. glass of wine, or one 1 oz. shot of 100 proof liquor

### Materials

#### Demographics and Gambling History

This questionnaire assessed demographic information such as age, sex, race, marital status, education level, and income. Gambling frequency, duration of gambling episodes, and alcohol consumption while gambling were also assessed.

#### The Daily Drinking Questionnaire (DDQ)

The DDQ evaluated typical and heaviest weekly drinking quantity and frequency during the past 30 days (Collins et al., 1985). The DDQ contains strong intercorrelation between collateral (*r* = .69) and self-report (*r* = .72) in assessing alcohol use among college students (Kivlahan et al., 1990). Four scores were derived from the DDQ and consists of the number of drinking days per average drinking week, average number of drinks per drinking day on an average week, number of drinking days per heaviest drinking week, and the average number of drinks per heaviest drinking week.

#### Alcohol Use Disorder Identification Test (AUDIT)

This 10-item self-report instrument identifies the extent to which individuals are experiencing negative consequences from drinking (Saunders et al., 1993). The AUDIT has been shown to have good internal consistency (*M*_α_ = 0.80; see De Meneses-Gaya et al., 2009 for a review of the psychometric properties of the measure).

#### Brief Assessment of Mood (BAM)

The BAM is a 6-item questionnaire designed to assess state level mood status and six mood state factors—Anxiety, Depression, Confusion, Anger, Vigor, and Fatigue (Whelan & Meyers, 2006). The BAM is based on the Profile of Mood States (POMS; McNair et al., 1971) but requires less than 30 s to administer, rapidly assessing changes in mood status or emotional response. For each item, individuals report how they feel ‘right now’ on a 5-point intensity scale. The BAM total mood disturbance score (BTMD) is calculated by summing the 6 BAM items after inverting the vigor rating. The BTMD has high levels of internal consistency (α = 0.85) and is highly correlated with the POMS Total Mood Disturbance Score (*r* = .88; Shearer et al., 2015). The vigor/energetic item of the BAM serves as an indicator of positive mood state and was used in analyses. The other items from the BAM collectively measure the presence or absence of negative mood state and are best assessed with the BTMD.

#### Alcohol Urge Questionnaire (AUQ)

The AUQ is a unidimensional 8-item questionnaire (Bohn et al., 1995) that assesses current cravings to consume alcohol. Subjects rated their level of agreement on statements about the desire to drink on a 7-point Likert scale. The AUQ has shown acceptable internal consistency (α = 0.75; LaRowe et al., 2020) and acceptable psychometric properties across multiple indices (Drummond & Phillips, 2002). The total craving score is calculated by summing the ratings for the eight items.

*Spielberger StateTrait Anxiety Inventory—StateAnxiety Portion (STAI)*.

The STAI is a 20-item questionnaire designed to measure the current level of subjective anxiety (Spielberger et al., 1970). Individuals answered how they feel ‘right now, at the moment’ on a 1 to 4 scale. Scores range from 10 to 40 with higher scores indicating greater levels of state-anxiety. Strong internal consistency has been observed among the items comprising the state subscale of the STAI (α = 0.94; Thomas & Cassady, 2021).

#### PostTest Alcohol Assessment

To further assess desire to consume alcohol, the experimenter asked participants: (1) Would you like to have an alcoholic drink? If the participant responded yes, (2) How many would you like?

### Procedure

All experimental and recruitment procedures were approved by the university’s Institutional Review Board. Prospective participants volunteered to participate via a posting on the university’s psychology department research website, which included a brief description of the experiment, inclusion and exclusion criteria for participation, and a statement noting that participants would have the opportunity to win prizes. Prospective participants were informed that if they were enrolled in a psychology class, they could also earn research credits. Eligible participants were randomly assigned to neutral, winning, or losing gambling or the movie watching condition using a table of randomly generated numbers.

Trained research assistants carried out all data collection procedures. Data collection sessions occurred during the afternoon and evening, time periods in which alcohol consumption may typically occur. Following arrival at the lab, participants were presented with an informed consent form and given the opportunity to ask any questions they may have. Following the provision of consent, participants completed the demographic and gambling questionnaire, DDQ, and AUDIT. After which, participants assigned to the gambling condition were taken to the gambling room, and those assigned to the movie condition were taken to the room where the movie was shown.

### Gambling Condition

The gambling room was decorated to approximate a casino setting with brightly colored and patterned carpet, colored lighting, a small bar, other casino-like decorations, and three modified slot machines with casino slot machine stools. Signs showing prizes offered to winning participants were posted on the walls and a soundtrack of casino sounds was played in the background. The modified slot machines consisted of a computer running a slot machine game designed to resemble one found in a casino installed within a genuine slot machine exterior. This approach allowed researchers to control the win/loss pattern of the game and record the gambling behaviors. The three slot machines were configured identically, so whichever machine was chosen provided the same experience for the participant. While playing, participants viewed a screen that included three spinning reels, the current balance of credits the player had remaining, and a chart displaying the possible winning combinations. Players bet up to three credits by pushing “bet one” or “bet max” buttons and then began the game by pushing a “spin” button. After initiating a spin, the game provided the spin animation and returned the predetermined outcome, alerting players if they had won on the screen with flashing lights and sounds. Players could immediately begin playing again by making another bet.

Slot machines in this study were pre-configured to provide a winning, losing, or neutral experience like that found at a casino. To create the three gambling conditions, an average payout found typically on casino slot machines of 92% was first used as a baseline. A pattern of wins and losses and payout multipliers was programmed to determine the outcome of the three game conditions. For example, spin 1 was set to yield a win returning “cherry, bell, cherry” symbols on the screen and a payout of 2 times the amount bet by the player; spin 2 was set to yield a loss with “bell, orange, lemon” symbols displayed and no payout. A unique pattern of wins and losses was created for each of the three gambling conditions so that participants would have a winning, losing, or neutral gambling experience depending on their assigned condition. Because players had the option of betting either 1, 2, or 3 credits per spin as is typical of casino slot machines, total credit amount could not be exactly controlled within the game. However, the experience of playing (and hence winning or losing), including the end result, would be consistent for each group. Preliminary pilot testing was carried out using regular slot machine players and a secondary sample of students to determine approximate payout percentages for each condition. Based on these trials, the winning condition was set to payout 300%, the losing condition 45%, and the neutral condition 95%.

Upon entering the gaming room, participants were invited to choose one of the three slot machines and instructed that they would gamble with 250 credits of imaginary currency. They were then informed that credits remaining at the end of the experiment would be exchanged for a proportional number of raffle tickets for several prizes, including gift certificates to local stores and restaurants. Participants were then told to play their selected slot machine for as long as they wished but for at least 30 min. The experimenter then left the room. After 20 min, the experimenter stopped the session and told the participant that they would be taking a break for a minute to complete a few questionnaires. This step was done to ensure participants were not anticipating the end of the experiment and possibly modifying their gambling as such.

### Movie Condition

Participants in the movie condition were led to a room containing a chair and a television and instructed to watch a movie (*The Saint*, 1997). The movie, and the specific scene shown, was chosen for its non-exciting content. After 20 min of viewing, the experimenter stopped the session and notified the participant that they would take a short break to complete a few questionnaires.

### Concluding Procedure

Following the experimental manipulation to which participants were assigned, the BAM, AUQ, and STAI state-anxiety measures were completed in either the gambling or movie room. Participants also responded to two hypothetical questions, “If alcohol were available, would you like to have a drink?” If a participant responded yes, they were then asked, “How many drinks would you like?” Following these hypothetical questions, participants were debriefed on the nature of the experiment, provided with the researchers’ contact information, and provided an informational sheet with information about alcohol and gambling related harms. All participants were then provided prize raffle tickets. Those in the gambling condition received tickets based on their performance on the slot machine program, while those in the movie condition received a set number of tickets.

## Results

### Evaluation of Potential Covariates

Analyses were performed to examine demographic, drinking, and gambling behaviors as potential covariates. These included age, gender, years of education, income, past year gambling frequency, report of drinking while gambling, AUDIT score, average and heaviest weekly alcohol consumption in the past month. Pearson’s correlations were calculated between the potential covariates, and the dependent variables and covariates with a significance level of *p* < .05 were retained for the final analysis. Average weekly alcohol consumption was significantly related to AUQ score (*r* = .47), wanting a drink after participation (*r* = .40), and number of drinks wanted after participation (*r* = .42, *p* < .001), and was therefore considered as a covariate in the analysis of urge to drink.

### Impact of Activity Condition on Mood State

To examine the hypothesis that condition assignment would influence mood state, separate Analysis of Variance (ANOVA) models were computed comparing STAI state-anxiety, BMTD, and the vigor subscale score across the neutral gambling, winning gambling, losing gambling, and movie conditions. No significant effects were revealed for STAI state-anxiety (*F*(2, 72) = 1.33, *p* = .27), BMTD (*F*(2, 72) = 1.69, *p* = .18), or vigor scores (*F*(2, 72) = 0.73, *p* = .54).

### Impact of Activity Condition on Urge to Drink

**Fig. 1:**
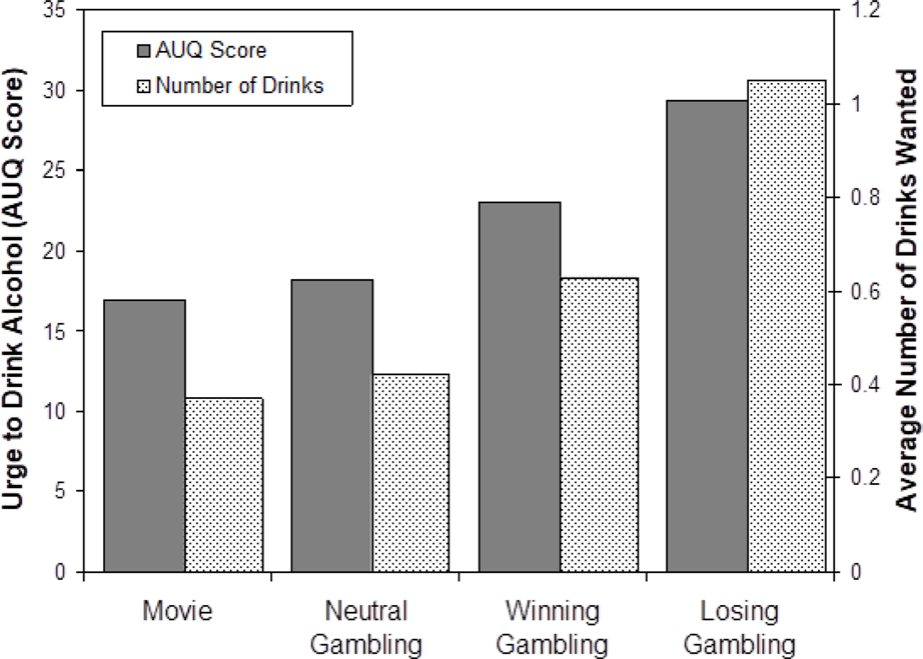
Mean urge to drink alcohol scores and number of alcoholic drinks wanted following activity participation (neutral slot machine gambling, winning slot machine gambling, losing slot machine gambling, watching movie control). AUQ Score = Alcohol Urge Questionnaire; Number of Drinks = Number of Drinks Wanted After Participation

An Analysis of Covariance (ANCOVA) was conducted comparing urge to drink alcohol across the four conditions. There was a significant effect of condition on urge to consume alcohol while holding average weekly alcohol consumption in the past month constant, *F*(3, 71) = 5.13, *p* < .01. Follow-up contrasts revealed that those in a losing gambling condition reported greater urges to consume alcohol than those who watched a movie (*p* < .001) and those in the neutral gambling condition (*p* < .005), but not compared to those in the winning gambling condition (*p* = .08). There was no significant difference between those in the winning condition and either the neutral gambling (*p* = .17) or movie conditions (*p* = .09). Additionally, there was no difference between the neutral gambling and the movie conditions (*p* = .75). See Fig. 1 for a graphical representation of variation in the number of drinks wanted after participation and AUQ as a function of group assignment.

Additional analysis was conducted on the two post-test questions to confirm that these questions assessed desire to drink alcohol. AUQ score was positively correlated with wanting a drink after participation (*r* = .50) and with number of drinks wanted after participating (*r* = .62). Additional analysis of urge to drink alcohol was conducted utilizing two ANCOVAs comparing the effect of conditions on the two post-test drinking questions while holding average weekly alcohol consumption in the past month constant. The first analysis examined the relation between participants that indicated that they wanted a drink after participating and those that did not across conditions. Results indicated no significant differences, *F*(3, 72) = 2.58, *p* = .06. The second ANCOVA examined number of drinks wanted after participating across conditions, which revealed a significant effect, *F*(3, 72) = 3.44, *p* < .05. Follow-up contrasts revealed that those in the losing condition reported wanting more drinks compared to those in the movie watching (*p* < .005) and neutral gambling conditions (*p* < .01) but not compared to those who gambled in a winning condition (*p* = .08). There was no significant difference between those in winning and neutral gambling (*p* = .38) or movie conditions (*p* = .09) or between those in the neutral gambling or movie conditions (*p* = .83).

### Relation Between Mood Measures and Urge to Drink

To attempt to clarify the relation between mood measures and desire to drink variables, Pearson correlations were performed across all conditions. STAI state-anxiety was shown to be related to urge to drink scores (AUQ; *r* = .30, *p* < .01) and to number of drinks wanted after participation (*r* = .27, *p* < .05). To further investigate the relation between mood state and urge to drink, the sample set was dichotomized into high and low urge to drink groups defined using a cutoff based on the median AUQ score of the entire sample set (median = 19.5). The resulting high urge group contained 38 participants (*M*_*AUQ*_ = 29.13, *SD*_*AUQ*_ = 11.77) and the low urge group contained 38 participants (*M*_*AUQ*_ = 14.61, *SD*_*AUQ*_ =6.50). Individual ANOVAs were then conducted comparing high and low urge with STAI state-anxiety, BTMD, and vigor. STAI state-anxiety (*F*(1, 74) = 5.80, *p* < .05) was significantly different while BTMD (*F*(1, 74) = 0.60, *p* = .44) and vigor (*F*(1, 74) = 0.17, *p* = .68) were not. Results indicated that those in the high urge group (*M* = 36.71, *SD* = 9.52) reported greater state-anxiety compared to those in the low urge group (*M* = 31.39, *SD* = 9.71).

## Discussion

This experiment used random assignment to condition to examine the effect of gambling on mood and the propensity to experience alcohol consumption urges. The first hypothesis was not supported as gambling did not significantly influence mood compared to watching a movie, an unexpected finding that directly contrasts previous research observing increases in positive mood states following positive gambling outcomes (Lipinski et al., 2023b; Mishra et al., 2010). Further, results were also inconsistent with existing literature pertaining to mood and gambling (e.g., Gee et al., 2005; Hills et al., 2001; Mishra et al., 2010; Wulfert et al., 2008). At the same time, the relation between gambling and mood is not definitive. Stewart and colleagues (2002) found that increases in negative mood states were only seen in those gamblers who chose to consume alcohol while gambling. Those who did not drink or participants who watched a movie did not show changes in negative mood. Further, when losses were statistically controlled for, this effect was eliminated. It is important to note that this study did not measure mood states after gambling wins directly and that doing so may have provided additional insight into the relation of positive gambling experiences and alcohol consumption urges.

Partially in support of the second hypothesis, those who gambled reported higher urges to drink alcohol compared to those who watched a movie. This finding is consistent with previous research demonstrating increased alcohol consumption (Tobias-Webb et al., 2019) and urges to consume alcohol (Lipinski et al., 2023b) following slot machine play. Importantly, it was found that significantly greater urges to drink and number of drinks desired at the end of the experimental period was observed among individuals who experienced losing gambling outcomes compared to those who experienced neutral outcomes or watched a movie. However, no significant differences in drinking urges or number of drinks desired were observed between those who experienced winning gambling outcomes and any other experimental condition. These findings provide further support for the relation between experiencing losing outcomes while gambling and urges to drink alcohol. When individuals experience losses while gambling, they may want to drink more. Previous research has observed that individuals who gamble regularly may expect that consuming alcohol while gambling will increase their skill, luck, and winnings (Horn et al., 2023). Therefore, the relation between gambling losses and the urge to consume alcohol may be potentiated among individuals who believe that alcohol consumption may increase their abilities while gambling. As such, future research may seek to examine the interaction between gambling outcomes and substance response expectancies on substance use behavior (e.g., cravings, drinking) while gambling.

Comparisons between mood and urge to drink measures were consistent with the findings of Lipinski and colleagues (2023b). Also consistent, individuals in the high urge to drink group reported greater state-anxiety compared to those in the low urge to drink group. This finding provides additional evidence that how individuals experienced the gambling activity contributed to desire to consume alcohol. Those who experienced greater anxiety while gambling were more likely to want a drink, regardless of winning or losing. This pattern is pertinent as anxiety and anticipation are inherent to wagering on an uncertain outcome. It may be that this anticipation can contribute to an overall feeling of anxiety in some individuals or in some gambling outcomes, which in turn may lead to greater urges to drink. Positive correlations between state-anxiety and alcohol consumption urges are consistent with previous research that linked negative mood state to alcohol consumption (Bresin et al., 2018). Losing and the stress experienced related to monetary loss likely contribute to greater subjective anxiety (e.g., Gee et al., 2005). However, as winners and losers were not significantly different in this study, results suggest that other factors beyond mood also influence the desire to drink. Such factors likely include learned responses, such as positive and negative expectancy effects related to gambling and conditioned responses to cues in the gambling environment. Previous experiences with alcohol may also contribute. For example, individuals may use alcohol as a coping strategy to minimize unpleasant feelings. This finding is consistent with existing literature in that losses during gambling may prime an individual to alleviate negative mood states induced by unfavorable gambling experiences (Devos et al., 2018; Rockloff et al., 2011).

The present study provided further insight into the relation between gambling and urges to consume alcohol; however, it is not without limitations. One potential limitation of the present research was that participants at no point had the opportunity to order or consume alcoholic beverages – limiting the collection of data regarding the effect of gambling outcomes on alcohol consumption. Nevertheless, the present study incorporated the use of validated a self-report measure of alcohol craving (Bohn et al., 1995), expanding the budding literature investigating the effects of gambling on alcohol use behavior without exposing participants to the various health risks associated with even low levels of alcohol consumption (Anderson et al., 2023). The present study employed a simulated gambling environment, allowing for greater experimental control of the gambling experience (i.e., winning vs. losing) and other potential confounds. However, experimental gambling contexts may not completely capture the complexities of in-person gambling venues. Future studies may seek to apply experience sampling methodologies, such as ecological momentary assessment, to capture nuanced within-person mood and alcohol craving data during and following gambling in natural settings. It would be important in subsequent studies to explore other constructs that mediate the gambling experience and the decision to consume alcohol. While the present focus on mood was informed by the literature, the singular focus was also a limitation of this study. Although participant perceptions of the gambling experience (i.e., winning or losing) were not formally evaluated, preliminary pilot testing informed the rationale for the expected function of gambling outcomes in eliciting the desired subjective experiences. Another possible limitation was excluding any volunteer who upon screening appeared to have a gambling problem. This decision limited the depth of our understanding about how gambling experiences impact the alcohol craving of individuals with gambling disorder. Lastly, the limited sample size for the current study presents another potential limitation. Several of our analyses failed to reach statistical significance, suggesting the effect sizes between these variables may have been smaller than initially anticipated and leaving analyses underpowered.

In conclusion, these findings indicate that experiencing gambling losses may increase the likelihood of gamblers experiencing alcohol consumption urges. Further, this study contributes to the existing literature by providing a deeper understanding of factors that influence alcohol consumption urges in the context of gambling. This study informs prevention and intervention efforts which may target co-occurring alcohol use and problematic gambling behaviors.

## Data Availability

The data that supported this study are available from the corresponding author, JPW, upon reasonable request.
